# Coordination of bis­(pyrazol-1-yl)amine to palladium(II): influence of the co-ligands and counter-ions on the mol­ecular and crystal structures^1^

**DOI:** 10.1107/S205698901402595X

**Published:** 2015-01-01

**Authors:** María de los Angeles Mendoza, Sylvain Bernès, Guillermo Mendoza-Díaz

**Affiliations:** a Departamento de Ingenierías Química Electrónica y Biomédica, División de Ciencias e Ingenierías, Campus León, Universidad de Guanajuato, Loma del Bosque 103, Lomas del Campestre, León, Guanajuato 37150, Mexico; b DEP Facultad de Ciencias Químicas, UANL, Guerrero y Progreso S/N, Col. Treviño, 64570 Monterrey, N.L., Mexico

**Keywords:** crystal structure, coordination compounds, bis­[2-(3,5-di­methyl­pyrazol-1-yl)eth­yl]amine (pza) ligand, bis­(pyrazol-1-yl)amine, palladium(II)

## Abstract

The structures of a series of complexes with general formula *n*[Pd(pza)*X*]*Y*·*m*H_2_O (*n* = 1, 2; *X* = Cl, Br, I, N_3_, NCS; *Y* = NO_3_, I, N_3_, [Pd(SCN)_4_]; *m* = 0, 0.5, 1) have been determined, where pza is the tridentate ligand bis­[2-(3,5-di­methyl­pyrazol-1-yl)eth­yl]amine, C_14_H_23_N_5_. In all complexes, {bis­[2-(3,5-di­methyl­pyrazol-1-yl-κ*N* ^2^)eth­yl]amine-κ*N*}chlorido­palladium nitrate, [Pd(pza)Cl]NO_3_, (1), {bis­[2-(3,5-di­methyl­pyrazol-1-yl-κ*N* ^2^)eth­yl]amine-κ*N*}bromido­palladium nitrate, [Pd(pza)Br]NO_3_, (2), {bis­[2-(3,5-di­methyl­pyrazol-1-yl-κ*N* ^2^)eth­yl]amine-κ*N*}iodido­palladium iodide hemihydrate, [Pd(pza)I]I·0.5H_2_O, (3), azido{bis­[2-(3,5-di­methyl­pyrazol-1-yl-κ*N* ^2^)eth­yl]amine-κ*N*}palladium azide monohydrate, [Pd(pza)N_3_]N_3_·H_2_O, (4), and bis­[{bis­[2-(3,5-di­methyl­pyrazol-1-yl-κ*N* ^2^)eth­yl]amine-κ*N*}(thio­cyanato-κ*N*)palladium] tetra­kis­(thio­cyanato-κ*S*)palladate, [Pd(pza)NCS]_2_[Pd(SCN)_4_], (5), the [Pd(pza)*X*]^+^ complex cation displays a square-planar coordination geometry, and the pza ligand is twisted, approximating twofold rotation symmetry. Although the pza ligand is found with the same conformation along the series, the dihedral angle between pyrazole rings depends on the co-ligand *X*. This angle span the range 79.0 (3)–88.6 (1)° for the studied complexes. In (3), two complex cations, two I^−^ anions and one water mol­ecule of crystallization are present in the asymmetric unit. In (5), the central amine group of pza is disordered over two positions [occupancy ratio 0.770 (18):0.230 (18)]. The complex [Pd(SCN)_4_]^2−^ anion of this compound exhibits inversion symmetry and shows the Pd^2+^ transition metal cation likewise in a square-planar coordination environment. Compound (5) is also a rare occurrence of a non-polymeric compound in which the pseudohalide ligand NCS^−^ behaves both as thio­cyanate and iso­thio­cyanate, *i.e.* is coordinating either through the N atom (in the cation) or the S atom (in the anion).

## Abstract 2

The crystal structures for five Pd^II^ complexes containing the tridentate ligand bis­[2-(3,5-di­methyl­pyrazol-1-yl)eth­yl]amine (pza) are reported. The co-ligand completing the square-planar coordination of the Pd^II^ centre influences the conformation of the pza ligand.

## Chemical context   

The coordination chemistry of transition metals having a *d* ^8^ shell is clearly dominated by the square-planar geometry, which gives strong crystal field stabilization, because filled orbitals *d*_z2_ and degenerated orbitals (*d*_xz_ *d*_yz_) do not inter­act directly with orbitals of the ligands. This holds true for group 10 metal complexes, for which the tetra­hedral geometry is considered as an oddity (Alvarez *et al.*, 2005 ▸).
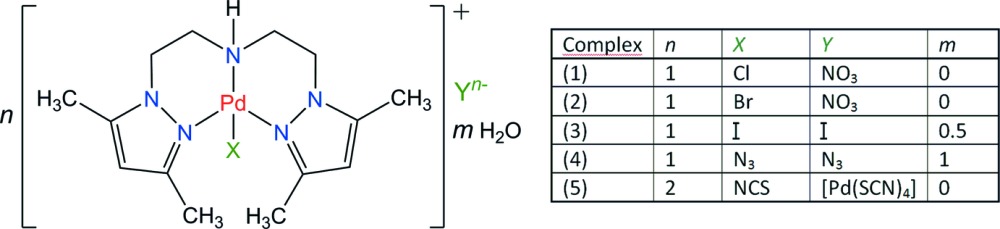


We synthesized a series of such square-planar complexes, with general formula *n*[Pd(pza)*X*]*Y*·*m*H_2_O, in which pza is the tridentate ligand bis-[2-(3,5-di­methyl­pyrazol-1-yl)eth­yl]amine, and *X*, *Y* are halide, pseudohalide, nitrate, or a complex anion. This series was first considered within a larger project related to a systematic study of modifications of *cis*-platin, obtained through the substitution of NH_3_ ligands by *N*-heterocyclic systems, like imidazole- and pyrazole-based ligands. The Pd^II^ synthetic chemistry may be easily transferred to Pt^II^, with the advantage that Pd^II^ starting materials are somewhat cheaper than their Pt^II^ analogues. On the other hand, regarding the chemical crystallography, Pd^II^ complexes are almost always isostructural to their Pt^II^ analogues. Finally, any new Pd^II^ complex is also of potential inter­est for studies about the fundamental aspects of the catalysis of the Heck reaction type.

We thus focused our efforts on the crystallographic characterization of the Pd^II^ complexes obtained as single crystals, with the hope of rationalizing the effect of the co-ligand *X* and counter-ion *Y* on the mol­ecular and crystal structures of the complex [Pd(pza)*X*]^+^ cations. An earlier report of the crystal structure of the starting material, [Pd(pza)Cl]Cl·2H_2_O has been given (Mendoza *et al.*, 2006 ▸), and we now report on the characterization of [Pd(pza)Cl]NO_3_ (1), [Pd(pza)Br]NO_3_ (2), [Pd(pza)I]I·0.5(H_2_O) (3), [Pd(pza)N_3_]N_3_·H_2_O (4), and 2[Pd(pza)NCS][Pd(SCN)_4_] (5).

## Structural commentary: mol­ecular and crystal structures   

**Figure 1:**
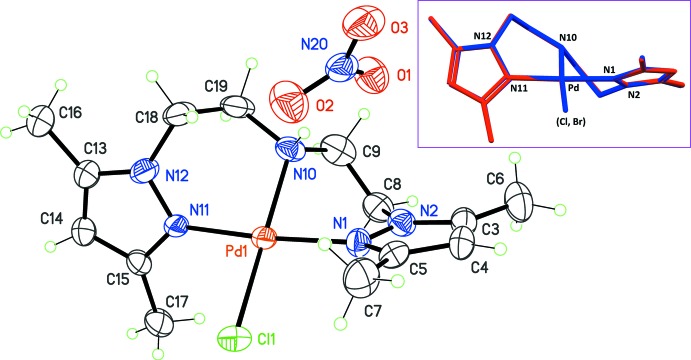
View of the mol­ecular structure of complex (1), corresponding to *X* = Cl^−^ and *Y* = NO_3_^−^, with displacement ellipsoids for non-H atoms drawn at the 30% probability level. The inset is an overlay (*Mercury*; Macrae *et al.*, 2008 ▸) of the cations in (1) and (2), in which *X* = Br^−^.

Complex (1) is a result of the substitution of the counter-ion *Y* = Cl^−^ in the starting material, *i.e.* in the dihydrate [Pd(pza)Cl]Cl·2H_2_O by a nitrate, but crystallizes as an anhydrous species, [Pd(pza)Cl]NO_3_ (Fig. 1 ▸). As expected, the square-planar coordination of the metal cation is retained, and the conformation of the pza ligand is not affected by the counter-ion substitution. The cation conformation may be characterized by the dihedral angle between the pyrazole mean planes, 85.1 (3)° *versus* 87.62 (11)° in the chloride salt (Mendoza *et al.*, 2006 ▸). A least-squares fit between the [Pd(pza)Cl]^+^ cations in the chloride and nitrate salts gives an r.m.s. deviation of 0.124 Å. However, the crystal structures are different because the water mol­ecules in the chloride dihydrate are determinant for the supra­molecular arrangement through hydrogen-bonding and inter­molecular contacts. In (1), the nitrate ion inter­acts with the central amine group of the pza ligand, with a N10—H10⋯O1 separation of 1.98 Å. Other inter-ion contacts beyond the asymmetric unit are unexceptional, and the observed crystal structure is basically a consequence of Coulombic inter­actions rather than hydrogen bonds (Table 1 ▸).

Complex (2), with *X* = Br^−^ and *Y* = NO_3_^−^ is isostructural with the *X* = Cl^−^ analogue (1). However, a slight relaxation of the folded pza ligand is observed, with a dihedral angle between pyrazole rings of 83.6 (2)°. An overlay between cations in (1) and (2) gives a small deviation of 0.049 Å (Fig. 1 ▸, inset). The nitrate anion inter­acts with the complex cation in (2) with a distance N10—H10⋯O1 = 1.98 Å (Table 2 ▸). Thus, the nature of the halogen co-ligand *X* in [Pd(pza)*X*]NO_3_ seems to be unimportant for the resulting crystal structure.

**Figure 2:**
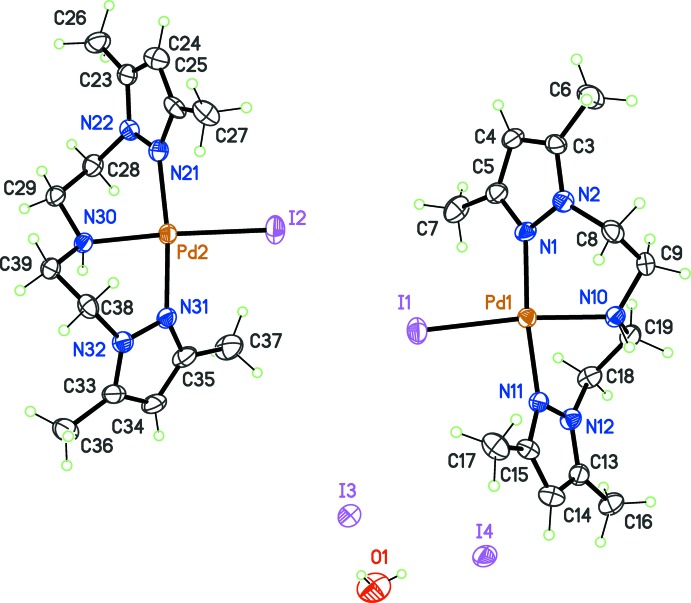
View of the mol­ecular structure of complex (3), corresponding to *X* = *Y* = I^−^, with displacement ellipsoids for non-H atoms drawn at the 30% probability level.

Complex (3), built up with *X* = *Y* = iodide, crystallized as a hemihydrate, with two cation complexes and two free iodide ions in the asymmetric unit (Fig. 2 ▸). The square-planar geometry of Pd^II^ is retained, as well as the pza conformation. However, the relaxation of folding, observed with *X* = Br^−^ in compound (2), is amplified with *X* = I^−^: the angle between the pyrazole rings is now 79.0 (3) and 83.3 (3)°, for the Pd1 and Pd2 cations, respectively. There seems to be a regular trend for [Pd(pza)*X*]^+^ cations: the smaller the ionic radius of the co-ligand *X*, the closer the angle between the pyrazole rings is to 90°. A possible rationalization of this observation is that methyl groups substituting pyrazole rings at position 3 inter­act with the co-ligand *X*. This destabilizing steric inter­action favors the twisting of pza, which in general adopts a non-crystallographic twofold rotation symmetry. However, the large iodide anion forces the separation between methyl groups, compared to the small chloride ion. In order to keep the coordination geometry around Pd^II^ as planar as possible, the heterocycles in pza then make a slight rotation motion, which is reflected in the deviation from orthogonality of these terminal rings. In other words, the combined twisting and folding motions of the pza ligand lead to as planar as possible a coordination environment for Pd^II^. Counter-ions *Y* and lattice water mol­ecules have only slight influences, if any, on the cation conformation. In the case of (3), the water mol­ecule behaves both as a donor and acceptor group for hydrogen bonding. O—H⋯I bonds are formed with the non-coordin­ating iodide anions, and the central amine group of pza forms a N—H⋯O bond with the same water mol­ecule (Table 3 ▸). However, as for previous complexes (1) and (2), no extended supra­molecular structures are formed in the crystal.

**Figure 3:**
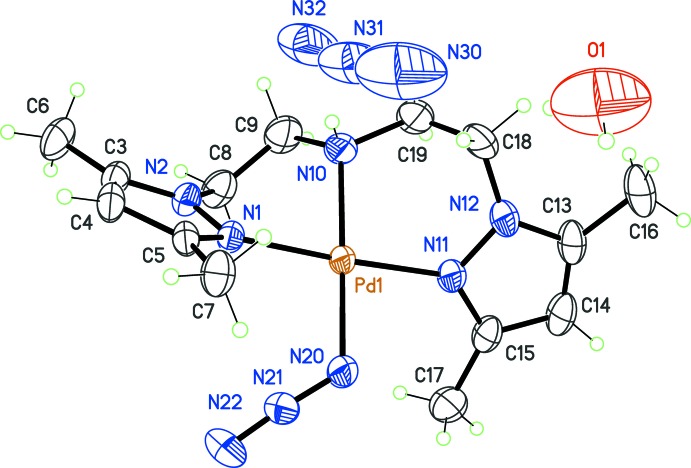
View of the mol­ecular structure of complex (4), corresponding to *X* = *Y* = N_3_^−^, with displacement ellipsoids for non-H atoms drawn at the 30% probability level.

Using the pseudohalide *X* = *Y* = azide, compound (4) was crystallized as an hydrate, [Pd(pza)N_3_]N_3_·H_2_O (Fig. 3 ▸). The nitro­gen atoms in the coordinating N_3_^−^ ligand are not steric­ally demanding as the iodide ion in (3) and, as a consequence, the pyrazole rings come back in a more orthogonal arrangement, identical to that observed in [Pd(pza)Cl]^+^. The dihedral angle between pyrazole rings is 87.3 (1)° in (4). The strongest hydrogen bond is found between the amine group of pza and the free azide ion, the N10—H10⋯N32 separation being 1.95 Å and the angle for the contact 171° (Table 4 ▸).

**Figure 4:**
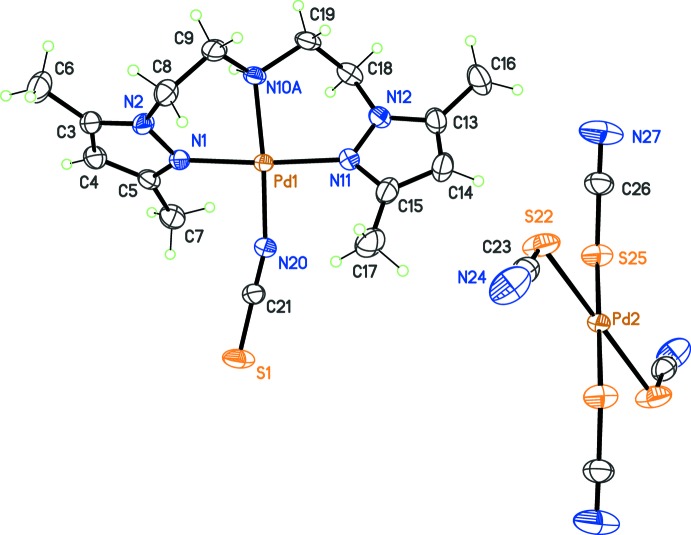
View of the mol­ecular structure of complex (5), corresponding to *X* = NCS^−^ and *Y* = [Pd(SCN)_4_]^2−^, with displacement ellipsoids for non-H atoms at the 30% probability level. Only one position for the disordered amine group in the cation has been retained (N10*A*). In the anion, unlabelled atoms are generated by symmetry code (−*x* + 1, −*y* + 2, −*z* + 2).

Finally, in the fifth compound (5), the counter-ion *Y* is a complex anion, namely [Pd(SCN)_4_]^2−^. The formula for (5) is 2[Pd(pza)NCS][Pd(SCN)_4_], and the anion is located about an inversion centre, while the cation is in a general position (Fig. 4 ▸). The pza ligand in [Pd(pza)NCS]^+^, in contrast to previous compounds, has the amine group N10 disordered over two positions, N10*A* and N10*B*, with occupancies 0.770 (18) and 0.230 (18), respectively. The same type of disorder was previously reported for an Au^III^ complex (Segapelo *et al.*, 2011 ▸). In spite of this disorder, the general conformation of pza is identical to that observed in compounds (1)–(4), approximating the non-crystallographic twofold rotation symmetry. The co-ligand *X* = NCS^−^ coordin­ates through its N atom, and the local environment of the metal is very similar to that resulting from azide coord­in­ation in complex (4). The dihedral angle between pyrazole rings should thus be close to 90°. The actual value is 88.6 (1)°. The anion [Pd(SCN)_4_]^2−^ is also square-planar, but with the ligands coordinating in a κ*S-*fashion, while in the cation, the NCS ligand is bound in a κ*N-*fashion to the metal cation. If complexes with bridging thio­cyanate ligands are not considered, very few structures are known in which the ambidentate ligand NCS^−^ is bonded in two modes (κ*S*- and κ*N*-) to the same transition metal. In the case of Pd^II^, classified as a soft acid in the Pearson’s HSAB concept, the soft base SCN^−^ should have a preference for the κ*S*-coordination. Apparently, only a few non-polymeric crystal structures have been reported including both coordination modes of SCN^−^ to this metal (*e.g*. Paviglianiti *et al.*, 1989 ▸; Chang *et al.*, 2005 ▸). In the crystal structure, weak hydrogen bonds between the disordered amino group and the NCS groups of neighbouring cations and anions are observed (Table 5 ▸).

## Database survey   

The ligand pza has been widely used in coordination chemistry. The current release of the CSD (Version 5.35 with all updates; Groom & Allen, 2014 ▸) affords 39 entries distributed over 18 articles. With Pd^II^, two structures are reported to date, which are pseudopolymorphs with *X* = *Y* = Cl^−^ (Mendoza *et al.*, 2006 ▸; Guzei *et al.*, 2010 ▸). Other transition metals have been coordinated by pza and structures are available for Co^II^ (van Berkel *et al.*, 1994 ▸; Massoud *et al.*, 2012*a* ▸, 2013 ▸), Ni^II^ (Ajellal *et al.*, 2006 ▸; Massoud *et al.*, 2012*a* ▸, 2013 ▸), Cu^II^ (van Berkel *et al.*, 1994 ▸; Martens *et al.*, 1995 ▸; Kim *et al.*, 2000 ▸; Monzani *et al.*, 2000 ▸; Riklin *et al.*, 2001 ▸; Massoud *et al.*, 2012*a* ▸,*b* ▸, 2013 ▸), Zn^II^ (Burth & Vahrenkamp, 1998 ▸; Lian *et al.*, 2007*a* ▸; Lee *et al.*, 2007 ▸; Massoud *et al.*, 2013 ▸), Cd^II^ (Griffith *et al.*, 1987 ▸; Massoud *et al.*, 2013 ▸), Re^I^ (Alves *et al.*, 2002 ▸) and Au^III^ (Segapelo *et al.*, 2011 ▸). The pza ligand generally behaves as a tridentate ligand, with exceptions for some Zn^II^ compounds, in which one pyrazole ring is not coordinating to the metal (Burth & Vahrenkamp, 1998 ▸; Lian *et al.*, 2007*a* ▸; Lee *et al.*, 2007 ▸). Few complexes have also been prepared with *s*- and *p*-metals, *viz*. Li^I^ (Lian *et al.*, 2007*a* ▸), Mg^II^ (Lian *et al.*, 2007*b* ▸), and Al^III^ (Lian *et al.*, 2007*a* ▸).

The conformation observed for pza is determined by the coordination number of the metal centre. For example, hexa-coordinated transition metals like Ni^II^ or Cd^II^ favor the facial coordination of pza, which is then found in a folded conformation, while coordination numbers 5 and 4 promote some defolding. The ligand pza with the dihedral angle between pyrazole rings very close to 0° has been observed in Co^II^ complexes (Massoud *et al.*, 2012*a* ▸, 2013 ▸). A conformation for pza close to that observed in (1)–(5) has been reported with Mg^II^ (Lian *et al.*, 2007*b* ▸) and Au^III^ (Segapelo *et al.*, 2011 ▸).

## Synthesis and crystallization   

**Figure 5:**
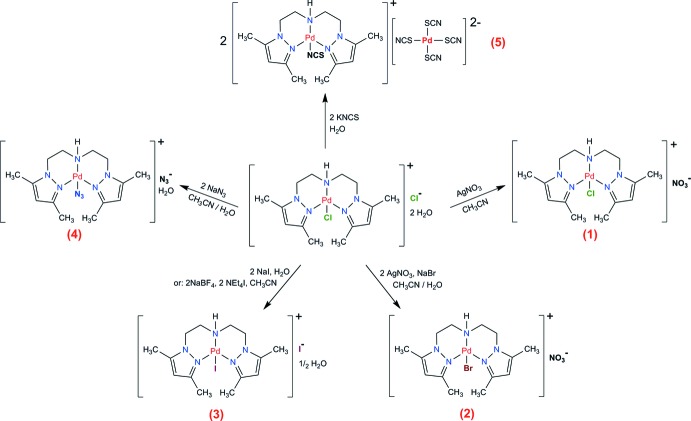
General synthetic scheme for complexes (1)–(5).

Complexes (1)–(5) were synthesized starting from [Pd(pza)Cl]Cl·2H_2_O (Mendoza *et al.*, 2006 ▸), by substitution of co-ligands and counter-ions, as depicted in Fig. 5 ▸.

**Synthesis of (1).** [Pd(pza)Cl]Cl·2H_2_O (1 mmol) was dissolved in CH_3_CN, and a solution of AgNO_3_ (1 mmol in CH_3_CN) was added slowly. The mixture was stirred for 1 h at room temperature. After elimination by filtration of the white precipitate of AgCl, the mixture was further stirred for 1 h. Evaporation of the solvent afforded complex (1) as a brown–yellow solid, in 82% yield, and crystals were obtained by recrystallization from CH_3_CN.

**Synthesis of (2).** [Pd(pza)Cl]Cl·2H_2_O (1 mmol) was dissolved in CH_3_CN, and a solution of AgNO_3_ (2 mmol in CH_3_CN) was added slowly. The mixture was stirred for 2 h at room temperature, and the precipitated AgCl was removed by filtration. An aqueous solution of NaBr (1 mmol) was then added, and NaNO_3_ precipitates, which was removed by filtration. The solution was further stirred for 5 h. Evaporation of the solvent afforded complex (2) as a yellow solid, in 76% yield, and crystals were obtained by recrystallization from CH_3_CN.

**Synthesis of (3).** [Pd(pza)Cl]Cl·2H_2_O (1 mmol) was dissolved in CH_3_CN (5 ml) and a solution of 2 mmol of NaBF_4_ in CH_3_CN was added slowly. After elimination of NaCl by filtration, a solution of 2 mmol of NEt_4_I in CH_3_CN was added slowly, and the mixture, which turned red, was stirred for 6 h at room temperature. Evaporation of the solvent afforded complex (3) as a red solid, in 82% yield, and crystals were obtained by recrystallization from CH_3_CN. Alternatively, complex (3) may be obtained in 89% yield by reacting an aqueous solution of [Pd(pza)Cl]Cl·2H_2_O (1 mmol) and NaI (2 mmol) for 6 h at room temperature.

**Synthesis of (4).** [Pd(pza)Cl]Cl·2H_2_O (1 mmol) was dissolved in CH_3_CN. A solution of NaN_3_ (2 mmol, CH_3_CN/H_2_O mixture 4:1, *v*/*v*) was added slowly. The formed precipitate of NaCl was eliminated by filtration, and the mixture was further stirred at room temperature for 10 h. Evaporation of the solvent afforded complex (4) as a yellow solid, in 61% yield, and crystals were obtained by recrystallization from CH_3_CN.

**Synthesis of (5).** [Pd(pza)Cl]Cl·2H_2_O (1 mmol) was dissolved in H_2_O, and an aqueous solution of 2 mmol of KNCS was added slowly. The mixture was stirred for 10 h at room temperature. The formed pink solid, (5), was separated by filtration and dried in reduced pressure at 313 K. Yield: 48%. Crystals were obtained by recrystallization from a mixture of CH_3_CN and CH_2_Cl_2_ (2:1, *v*/*v*).

## Refinement   

Crystal data, data collection and structure refinement details for (1)–(5) are summarized in Table 6 ▸. Data collection and refinement are routine works, except for a positional disorder found in (5) for sites N10*A*/N10*B*, for which the s.o.f. converged to 0.770 (18) and 0.230 (18), respectively. All H atoms bonded to C and N atoms were placed in calculated positions and refined as riding atoms, with fixed bond lengths of 0.93, 0.96, 0.97, and 0.90 Å for aromatic, methyl, methyl­ene, and amine groups, respectively. In (3) and (4), H atoms for water mol­ecules were found in difference maps, and first refined with free coordinates and restrained distances O—H = 0.85 (2) and H⋯H = 1.34 (4) Å. In the final cycles, water H atoms were fixed and refined as riding atoms. Isotropic displacement parameters for all H atoms were calculated as *U*_iso_(H) = *xU*_eq_(carrier atom), with *x* = 1.2 (methyl­ene, aromatic, and amine groups) or *x* = 1.5 (methyl and water).

## Supplementary Material

Crystal structure: contains datablock(s) 1, 2, 3, 4, 5, global. DOI: 10.1107/S205698901402595X/wm5076sup1.cif

Structure factors: contains datablock(s) 1. DOI: 10.1107/S205698901402595X/wm50761sup2.hkl

Structure factors: contains datablock(s) 2. DOI: 10.1107/S205698901402595X/wm50762sup3.hkl

Structure factors: contains datablock(s) 3. DOI: 10.1107/S205698901402595X/wm50763sup4.hkl

Structure factors: contains datablock(s) 4. DOI: 10.1107/S205698901402595X/wm50764sup5.hkl

Structure factors: contains datablock(s) 5. DOI: 10.1107/S205698901402595X/wm50765sup6.hkl

CCDC references: 1036262, 1036261, 1036260, 1036259, 1036258

Additional supporting information:  crystallographic information; 3D view; checkCIF report

## Figures and Tables

**Table 1:** Hydrogen-bond geometry (, ) for (1)

*D*H*A*	*D*H	H*A*	*D* *A*	*D*H*A*
C4H4*A*N20^i^	0.93	2.66	3.510(10)	153
C7H7*C*Cl1	0.96	2.81	3.410(10)	121
C8H8*A*O3^ii^	0.97	2.28	3.222(10)	163
N10H10N20	0.90	2.57	3.453(10)	167
N10H10O1	0.90	1.98	2.857(9)	164
N10H10O2	0.90	2.45	3.186(10)	140
C14H14*A*Cl1^iii^	0.93	2.82	3.629(9)	146
C16H16*A*O1^iv^	0.96	2.64	3.572(12)	164
C17H17*A*O3^ii^	0.96	2.53	3.491(12)	175
C17H17*C*Cl1	0.96	2.79	3.367(10)	119
C18H18*A*O2	0.97	2.51	3.364(11)	146
C18H18*B*O1^iv^	0.97	2.61	3.428(11)	142
C19H19*A*O3^iv^	0.97	2.47	3.112(11)	124

Symmetry codes: (i) 
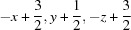
; (ii) 
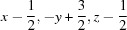
; (iii) 
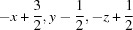
; (iv) 
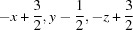
.

**Table 2:** Hydrogen-bond geometry (, ) for (2)

*D*H*A*	*D*H	H*A*	*D* *A*	*D*H*A*
C4H4*A*N20^i^	0.93	2.66	3.540(7)	159
C7H7*C*Br1	0.96	3.06	3.500(8)	110
C8H8*A*O3^ii^	0.97	2.30	3.219(9)	157
N10H10N20	0.90	2.54	3.427(6)	169
N10H10O1	0.90	1.98	2.857(7)	166
N10H10O2	0.90	2.43	3.181(8)	142
C14H14*A*Br1^iii^	0.93	2.88	3.687(6)	146
C16H16*A*O1^iv^	0.96	2.65	3.511(10)	150
C17H17*A*O3^ii^	0.96	2.55	3.485(10)	164
C17H17*C*Br1	0.96	2.98	3.459(8)	112
C18H18*A*O2	0.97	2.52	3.358(9)	144
C18H18*B*O1^iv^	0.97	2.65	3.468(8)	142
C19H19*B*O3^iv^	0.97	2.47	3.099(9)	122

Symmetry codes: (i) 
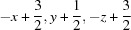
; (ii) 
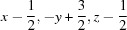
; (iii) 
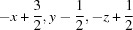
; (iv) 
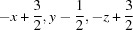
.

**Table 3:** Hydrogen-bond geometry (, ) for (3)

*D*H*A*	*D*H	H*A*	*D* *A*	*D*H*A*
O1H1I3	0.85	2.68	3.497(7)	161
O1H2I4	0.85	2.66	3.443(10)	155
N10H10*A*I3^i^	0.90	2.94	3.653(6)	137
N30H30*A*O1^ii^	0.90	2.22	3.011(9)	146
N30H30*A*I4^ii^	0.90	3.30	3.853(6)	122

Symmetry codes: (i) 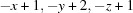; (ii) 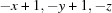.

**Table 4:** Hydrogen-bond geometry (, ) for (4)

*D*H*A*	*D*H	H*A*	*D* *A*	*D*H*A*
N10H10N32	0.90	1.95	2.838(11)	171
N10H10N31	0.90	2.66	3.460(13)	148
O1H11N32^i^	0.84	2.67	3.295(19)	132
O1H12N30	0.85	2.38	3.08(2)	140

Symmetry code: (i) 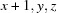.

**Table 5:** Hydrogen-bond geometry (, ) for (5)

*D*H*A*	*D*H	H*A*	*D* *A*	*D*H*A*
N10*A*H10*A*N24^i^	0.90	2.01	2.889(9)	166
N10*B*H10*B*S1^ii^	0.90	2.71	3.52(2)	151

Symmetry codes: (i) 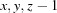; (ii) 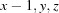.

**Table 6:** Experimental details

	(1)	(2)	(3)	(4)	(5)
Crystal data					
Chemical formula	[PdCl(C_14_H_23_N_5_)]NO_3_	[PdBr(C_14_H_23_N_5_)]NO_3_	[PdI(C_14_H_2_N_5_)]I0.5H_2_O	[Pd(N_3_)(C_14_H_23_N_5_)]N_3_H_2_O	[Pd(NCS)(C_14_H_23_N_5_)]_2_[Pd(NCS)_4_]
*M*_r_	465.23	509.69	630.58	469.85	1190.43
Crystal system, space group	Monoclinic, *P*2_1_/*n*	Monoclinic, *P*2_1_/*n*	Triclinic, *P* 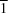	Monoclinic, *P*2_1_/*c*	Triclinic, *P* 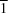
Temperature (K)	298	298	299	296	298
*a*, *b*, *c* ()	11.046(2), 12.2941(15), 14.0978(16)	10.934(6), 12.443(4), 14.112(6)	12.013(4), 12.089(4), 15.162(5)	8.132(3), 22.851(5), 11.372(3)	9.0286(17), 10.532(2), 13.066(3)
, , ()	90, 94.740(16), 90	90, 94.76(4), 90	106.17(2), 97.34(3), 106.79(3)	90, 109.03(2), 90	94.838(14), 100.947(12), 103.989(13)
*V* (^3^)	1907.9(5)	1913.4(14)	1972.0(11)	1997.8(10)	1172.5(4)
*Z*	4	4	4	4	1
Radiation type	Mo *K*	Mo *K*	Mo *K*	Mo *K*	Mo *K*
(mm^1^)	1.14	3.08	4.08	0.96	1.45
Crystal size (mm)	0.40 0.12 0.10	0.60 0.40 0.18	0.20 0.15 0.04	0.50 0.40 0.40	0.40 0.40 0.12

Data collection					
Diffractometer	Siemens P4	Siemens P4	Siemens P4	Siemens P4	Siemens P4
Absorption correction	scan (*XSCANS*; Siemens, 1996 ▸)	scan (*XSCANS*; Siemens, 1996 ▸)	scan (*XSCANS*; Siemens, 1996 ▸)	scan (*XSCANS*; Siemens, 1996 ▸)	scan (*XSCANS*; Siemens, 1996 ▸)
*T*_min_, *T*_max_	0.469, 0.517	0.206, 0.352	0.446, 0.523	0.266, 0.366	0.256, 0.378
No. of measured, independent and observed [*I* > 2(*I*)] reflections	4513, 3372, 2110	12224, 4962, 3329	8975, 6835, 4559	8431, 4032, 3528	8889, 5367, 4874
*R*_int_	0.044	0.080	0.043	0.056	0.038
(sin /)_max_ (^1^)	0.596	0.677	0.595	0.623	0.650

Refinement					
*R*[*F* ^2^ > 2(*F* ^2^)], *wR*(*F* ^2^), *S*	0.060, 0.158, 1.05	0.051, 0.148, 1.05	0.040, 0.101, 1.03	0.036, 0.097, 1.08	0.039, 0.107, 1.06
No. of reflections	3372	4962	6835	4032	5367
No. of parameters	230	231	414	248	282
H-atom treatment	H-atom parameters constrained	H-atom parameters constrained	H-atom parameters constrained	H-atom parameters constrained	H-atom parameters constrained
_max_, _min_ (e ^3^)	1.45, 1.11	1.10, 1.01	0.85, 1.04	0.55, 1.04	0.83, 1.06

Computer programs: *XSCANS* (Siemens, 1996 ▸), *SHELXS2014*, *SHELXL2014* and *SHELXTL* (Sheldrick, 2008 ▸) and *Mercury* (Macrae *et al.*, 2008 ▸).

## supplementary crystallographic information

### Crystal data

**Table:**

[Pd(NCS)(C_14_H_23_N_5_)]_2_[Pd(NCS)_4_]	*Z* = 1
*M**_r_* = 1190.43	*F*(000) = 596
Triclinic, *P*1	*D*_x_ = 1.686 Mg m^−^^3^
*a* = 9.0286 (17) Å	Mo *K*α radiation, λ = 0.71073 Å
*b* = 10.532 (2) Å	Cell parameters from 75 reflections
*c* = 13.066 (3) Å	θ = 4.7–12.4°
α = 94.838 (14)°	µ = 1.45 mm^−^^1^
β = 100.947 (12)°	*T* = 298 K
γ = 103.989 (13)°	Irregular Plate, pink
*V* = 1172.5 (4) Å^3^	0.40 × 0.40 × 0.12 mm

### Data collection

**Table:**

Siemens P4 diffractometer	4874 reflections with *I* > 2σ(*I*)
Radiation source: fine-focus sealed tube, FN4	*R*_int_ = 0.038
Graphite monochromator	θ_max_ = 27.5°, θ_min_ = 2.0°
2θ/ω scans	*h* = −11→6
Absorption correction: ψ scan (*XSCANS*; Siemens, 1996)	*k* = −13→13
*T*_min_ = 0.256, *T*_max_ = 0.378	*l* = −16→16
8889 measured reflections	3 standard reflections every 97 reflections
5367 independent reflections	intensity decay: 1%

### Refinement

**Table:**

Refinement on *F*^2^	Primary atom site location: structure-invariant direct methods
Least-squares matrix: full	Secondary atom site location: difference Fourier map
*R*[*F*^2^ > 2σ(*F*^2^)] = 0.039	Hydrogen site location: inferred from neighbouring sites
*wR*(*F*^2^) = 0.107	H-atom parameters constrained
*S* = 1.06	*w* = 1/[σ^2^(*F*_o_^2^) + (0.0569*P*)^2^ + 1.1014*P*] where *P* = (*F*_o_^2^ + 2*F*_c_^2^)/3
5367 reflections	(Δ/σ)_max_ < 0.001
282 parameters	Δρ_max_ = 0.83 e Å^−^^3^
0 restraints	Δρ_min_ = −1.06 e Å^−^^3^
0 constraints	

### Fractional atomic coordinates and isotropic or equivalent isotropic displacement parameters (Å^2^)

**Table:**

	*x*	*y*	*z*	*U*_iso_*/*U*_eq_	Occ. (<1)
Pd1	0.36057 (3)	0.61306 (2)	0.33358 (2)	0.03862 (9)	
S1	0.88007 (13)	0.60961 (16)	0.47191 (8)	0.0735 (4)	
N1	0.3690 (3)	0.4656 (3)	0.2291 (2)	0.0420 (6)	
N2	0.2424 (3)	0.3584 (3)	0.2003 (2)	0.0453 (6)	
C3	0.2603 (4)	0.2765 (4)	0.1225 (3)	0.0526 (8)	
C4	0.4009 (5)	0.3330 (4)	0.0994 (3)	0.0545 (9)	
H4A	0.4443	0.2987	0.0479	0.065*	
C5	0.4667 (4)	0.4500 (4)	0.1663 (2)	0.0448 (7)	
C6	0.1425 (6)	0.1487 (5)	0.0757 (4)	0.0814 (15)	
H6A	0.0469	0.1661	0.0415	0.122*	
H6B	0.1228	0.0957	0.1304	0.122*	
H6C	0.1822	0.1024	0.0253	0.122*	
C7	0.6181 (5)	0.5472 (5)	0.1716 (3)	0.0610 (9)	
H7A	0.6078	0.6349	0.1879	0.091*	
H7B	0.6475	0.5390	0.1050	0.091*	
H7C	0.6969	0.5308	0.2254	0.091*	
C8	0.1152 (4)	0.3501 (4)	0.2545 (3)	0.0531 (8)	
H8A	0.1555	0.3552	0.3295	0.064*	
H8B	0.0391	0.2653	0.2308	0.064*	
C9	0.0363 (4)	0.4580 (4)	0.2354 (3)	0.0564 (9)	
H9A	−0.0338	0.4356	0.1666	0.068*	0.770 (18)
H9B	−0.0264	0.4637	0.2874	0.068*	0.770 (18)
H9C	0.0179	0.4648	0.1608	0.068*	0.230 (18)
H9D	−0.0650	0.4328	0.2538	0.068*	0.230 (18)
N10A	0.1465 (5)	0.5881 (4)	0.2405 (5)	0.0423 (14)	0.770 (18)
H10A	0.1658	0.5870	0.1754	0.051*	0.770 (18)
N10B	0.1157 (15)	0.5806 (14)	0.2894 (18)	0.041 (4)	0.230 (18)
H10B	0.0858	0.5793	0.3513	0.049*	0.230 (18)
N11	0.3365 (3)	0.7611 (3)	0.4311 (2)	0.0450 (6)	
N12	0.2638 (3)	0.8493 (3)	0.3896 (2)	0.0490 (6)	
C13	0.2571 (5)	0.9392 (4)	0.4656 (4)	0.0641 (11)	
C14	0.3268 (6)	0.9078 (5)	0.5585 (4)	0.0698 (12)	
H14A	0.3399	0.9534	0.6251	0.084*	
C15	0.3743 (5)	0.7964 (4)	0.5356 (3)	0.0571 (9)	
C16	0.1801 (7)	1.0477 (6)	0.4450 (6)	0.0965 (19)	
H16A	0.2306	1.1016	0.3993	0.145*	
H16B	0.1883	1.1009	0.5102	0.145*	
H16C	0.0717	1.0105	0.4120	0.145*	
C17	0.4503 (7)	0.7208 (6)	0.6096 (3)	0.0769 (13)	
H17A	0.5170	0.6803	0.5766	0.115*	
H17B	0.3717	0.6535	0.6288	0.115*	
H17C	0.5115	0.7793	0.6716	0.115*	
C18	0.1923 (5)	0.8273 (4)	0.2784 (3)	0.0552 (9)	
H18A	0.2722	0.8288	0.2380	0.066*	
H18B	0.1442	0.8981	0.2613	0.066*	
C19	0.0713 (4)	0.6981 (4)	0.2488 (3)	0.0517 (8)	
H19A	0.0083	0.6857	0.3014	0.062*	0.770 (18)
H19B	0.0028	0.6979	0.1818	0.062*	0.770 (18)
H19C	0.0421	0.6799	0.1726	0.062*	0.230 (18)
H19D	−0.0209	0.7072	0.2734	0.062*	0.230 (18)
N20	0.5799 (4)	0.6337 (3)	0.4077 (3)	0.0558 (8)	
C21	0.7053 (4)	0.6233 (4)	0.4336 (3)	0.0517 (8)	
Pd2	0.5000	1.0000	1.0000	0.03984 (10)	
S22	0.27722 (13)	0.83780 (12)	0.91759 (10)	0.0736 (3)	
C23	0.2457 (4)	0.7188 (5)	0.9922 (3)	0.0567 (9)	
N24	0.2159 (6)	0.6323 (5)	1.0375 (3)	0.0849 (13)	
S25	0.45759 (12)	1.11365 (12)	0.85705 (8)	0.0604 (3)	
C26	0.2664 (5)	1.0806 (5)	0.8121 (3)	0.0617 (10)	
N27	0.1366 (6)	1.0626 (6)	0.7789 (4)	0.1036 (18)	

### Atomic displacement parameters (Å^2^)

**Table:**

	*U*^11^	*U*^22^	*U*^33^	*U*^12^	*U*^13^	*U*^23^
Pd1	0.03258 (13)	0.04589 (15)	0.03637 (13)	0.01607 (10)	0.00092 (9)	−0.00186 (9)
S1	0.0489 (5)	0.1354 (11)	0.0481 (5)	0.0505 (6)	0.0069 (4)	0.0103 (6)
N1	0.0391 (13)	0.0488 (15)	0.0379 (12)	0.0161 (11)	0.0053 (10)	−0.0014 (11)
N2	0.0382 (13)	0.0543 (16)	0.0404 (13)	0.0122 (12)	0.0071 (11)	−0.0062 (11)
C3	0.0485 (18)	0.060 (2)	0.0478 (17)	0.0214 (16)	0.0059 (14)	−0.0103 (15)
C4	0.051 (2)	0.069 (2)	0.0476 (18)	0.0254 (18)	0.0139 (15)	−0.0029 (16)
C5	0.0432 (16)	0.0583 (19)	0.0394 (15)	0.0249 (15)	0.0095 (12)	0.0067 (13)
C6	0.069 (3)	0.077 (3)	0.085 (3)	0.005 (2)	0.021 (2)	−0.031 (3)
C7	0.053 (2)	0.071 (3)	0.066 (2)	0.0196 (19)	0.0231 (18)	0.0106 (19)
C8	0.0507 (19)	0.058 (2)	0.0485 (18)	0.0084 (16)	0.0190 (15)	−0.0036 (15)
C9	0.0334 (16)	0.070 (2)	0.062 (2)	0.0129 (15)	0.0098 (15)	−0.0085 (18)
N10A	0.0321 (19)	0.059 (2)	0.038 (3)	0.0197 (16)	0.0053 (18)	0.0015 (17)
N10B	0.025 (5)	0.064 (8)	0.037 (9)	0.017 (5)	0.010 (5)	0.000 (6)
N11	0.0449 (14)	0.0451 (15)	0.0455 (14)	0.0178 (12)	0.0061 (11)	−0.0001 (11)
N12	0.0422 (14)	0.0464 (15)	0.0599 (17)	0.0171 (12)	0.0089 (12)	0.0043 (13)
C13	0.051 (2)	0.046 (2)	0.091 (3)	0.0135 (16)	0.012 (2)	−0.0113 (19)
C14	0.071 (3)	0.066 (3)	0.065 (2)	0.017 (2)	0.012 (2)	−0.021 (2)
C15	0.062 (2)	0.055 (2)	0.0474 (18)	0.0104 (17)	0.0080 (16)	−0.0063 (15)
C16	0.085 (4)	0.062 (3)	0.142 (5)	0.037 (3)	0.014 (4)	−0.015 (3)
C17	0.093 (4)	0.085 (3)	0.045 (2)	0.022 (3)	0.001 (2)	0.005 (2)
C18	0.0502 (19)	0.065 (2)	0.062 (2)	0.0296 (17)	0.0175 (16)	0.0199 (18)
C19	0.0364 (16)	0.075 (2)	0.0500 (18)	0.0282 (16)	0.0056 (13)	0.0091 (16)
N20	0.0388 (15)	0.0595 (19)	0.0648 (19)	0.0215 (13)	−0.0024 (13)	−0.0102 (15)
C21	0.0473 (19)	0.065 (2)	0.0425 (16)	0.0237 (16)	0.0014 (14)	−0.0029 (15)
Pd2	0.02961 (16)	0.0489 (2)	0.04031 (18)	0.01548 (13)	0.00172 (12)	0.00076 (14)
S22	0.0517 (6)	0.0667 (7)	0.0806 (7)	0.0003 (5)	−0.0215 (5)	0.0161 (5)
C23	0.0440 (18)	0.070 (2)	0.0495 (19)	0.0088 (17)	0.0082 (15)	−0.0040 (17)
N24	0.085 (3)	0.096 (3)	0.057 (2)	−0.007 (2)	0.015 (2)	0.016 (2)
S25	0.0460 (5)	0.0790 (7)	0.0549 (5)	0.0166 (4)	0.0031 (4)	0.0192 (5)
C26	0.056 (2)	0.081 (3)	0.051 (2)	0.032 (2)	0.0013 (17)	0.0122 (19)
N27	0.062 (3)	0.153 (5)	0.103 (4)	0.046 (3)	−0.001 (2)	0.044 (4)

### Geometric parameters (Å, º)

**Table:**

Pd1—N20	1.984 (3)	N10B—H10B	0.9000
Pd1—N1	2.005 (3)	N11—C15	1.340 (5)
Pd1—N11	2.009 (3)	N11—N12	1.353 (4)
Pd1—N10A	2.022 (4)	N12—C13	1.335 (5)
Pd1—N10B	2.111 (12)	N12—C18	1.447 (5)
S1—C21	1.607 (4)	C13—C14	1.362 (7)
N1—C5	1.342 (4)	C13—C16	1.493 (7)
N1—N2	1.365 (4)	C14—C15	1.372 (6)
N2—C3	1.336 (4)	C14—H14A	0.9300
N2—C8	1.449 (4)	C15—C17	1.478 (6)
C3—C4	1.366 (6)	C16—H16A	0.9600
C3—C6	1.496 (6)	C16—H16B	0.9600
C4—C5	1.379 (5)	C16—H16C	0.9600
C4—H4A	0.9300	C17—H17A	0.9600
C5—C7	1.482 (5)	C17—H17B	0.9600
C6—H6A	0.9600	C17—H17C	0.9600
C6—H6B	0.9600	C18—C19	1.493 (6)
C6—H6C	0.9600	C18—H18A	0.9700
C7—H7A	0.9600	C18—H18B	0.9700
C7—H7B	0.9600	C19—H19A	0.9700
C7—H7C	0.9600	C19—H19B	0.9700
C8—C9	1.494 (6)	C19—H19C	0.9700
C8—H8A	0.9700	C19—H19D	0.9700
C8—H8B	0.9700	N20—C21	1.153 (5)
C9—N10B	1.373 (14)	Pd2—S22	2.3085 (12)
C9—N10A	1.474 (6)	Pd2—S22^i^	2.3085 (12)
C9—H9A	0.9700	Pd2—S25	2.3227 (11)
C9—H9B	0.9700	Pd2—S25^i^	2.3227 (11)
C9—H9C	0.9700	S22—C23	1.656 (5)
C9—H9D	0.9700	C23—N24	1.133 (6)
N10A—C19	1.483 (5)	S25—C26	1.654 (4)
N10A—H10A	0.9000	C26—N27	1.133 (6)
N10B—C19	1.498 (15)		
			
N20—Pd1—N1	91.68 (12)	C19—N10B—Pd1	111.8 (9)
N20—Pd1—N11	92.64 (12)	C9—N10B—H10B	103.3
N1—Pd1—N11	175.60 (11)	C19—N10B—H10B	103.3
N20—Pd1—N10A	172.5 (2)	Pd1—N10B—H10B	103.3
N1—Pd1—N10A	82.54 (18)	C15—N11—N12	106.7 (3)
N11—Pd1—N10A	93.08 (18)	C15—N11—Pd1	134.6 (3)
N20—Pd1—N10B	166.4 (7)	N12—N11—Pd1	118.6 (2)
N1—Pd1—N10B	94.8 (5)	C13—N12—N11	110.3 (3)
N11—Pd1—N10B	81.2 (5)	C13—N12—C18	129.9 (3)
C5—N1—N2	106.4 (3)	N11—N12—C18	119.4 (3)
C5—N1—Pd1	134.7 (3)	N12—C13—C14	107.0 (4)
N2—N1—Pd1	118.4 (2)	N12—C13—C16	123.0 (5)
C3—N2—N1	110.7 (3)	C14—C13—C16	129.9 (5)
C3—N2—C8	130.8 (3)	C13—C14—C15	107.3 (4)
N1—N2—C8	118.5 (3)	C13—C14—H14A	126.4
N2—C3—C4	106.7 (3)	C15—C14—H14A	126.4
N2—C3—C6	123.2 (4)	N11—C15—C14	108.7 (4)
C4—C3—C6	130.1 (3)	N11—C15—C17	123.2 (4)
C3—C4—C5	107.6 (3)	C14—C15—C17	128.1 (4)
C3—C4—H4A	126.2	C13—C16—H16A	109.5
C5—C4—H4A	126.2	C13—C16—H16B	109.5
N1—C5—C4	108.6 (3)	H16A—C16—H16B	109.5
N1—C5—C7	123.2 (3)	C13—C16—H16C	109.5
C4—C5—C7	128.2 (3)	H16A—C16—H16C	109.5
C3—C6—H6A	109.5	H16B—C16—H16C	109.5
C3—C6—H6B	109.5	C15—C17—H17A	109.5
H6A—C6—H6B	109.5	C15—C17—H17B	109.5
C3—C6—H6C	109.5	H17A—C17—H17B	109.5
H6A—C6—H6C	109.5	C15—C17—H17C	109.5
H6B—C6—H6C	109.5	H17A—C17—H17C	109.5
C5—C7—H7A	109.5	H17B—C17—H17C	109.5
C5—C7—H7B	109.5	N12—C18—C19	111.4 (3)
H7A—C7—H7B	109.5	N12—C18—H18A	109.4
C5—C7—H7C	109.5	C19—C18—H18A	109.4
H7A—C7—H7C	109.5	N12—C18—H18B	109.4
H7B—C7—H7C	109.5	C19—C18—H18B	109.4
N2—C8—C9	112.1 (3)	H18A—C18—H18B	108.0
N2—C8—H8A	109.2	N10A—C19—C18	110.4 (3)
C9—C8—H8A	109.2	C18—C19—N10B	116.8 (5)
N2—C8—H8B	109.2	N10A—C19—H19A	109.6
C9—C8—H8B	109.2	C18—C19—H19A	109.6
H8A—C8—H8B	107.9	N10A—C19—H19B	109.6
N10B—C9—C8	115.9 (6)	C18—C19—H19B	109.6
N10A—C9—C8	113.3 (3)	H19A—C19—H19B	108.1
N10A—C9—H9A	108.9	C18—C19—H19C	108.1
C8—C9—H9A	108.9	N10B—C19—H19C	108.1
N10A—C9—H9B	108.9	C18—C19—H19D	108.1
C8—C9—H9B	108.9	N10B—C19—H19D	108.1
H9A—C9—H9B	107.7	H19C—C19—H19D	107.3
N10B—C9—H9C	108.3	C21—N20—Pd1	164.8 (3)
C8—C9—H9C	108.3	N20—C21—S1	179.0 (4)
N10B—C9—H9D	108.3	S22—Pd2—S22^i^	180.0
C8—C9—H9D	108.3	S22—Pd2—S25	87.77 (4)
H9C—C9—H9D	107.4	S22^i^—Pd2—S25	92.23 (4)
C9—N10A—C19	112.4 (4)	S22—Pd2—S25^i^	92.23 (4)
C9—N10A—Pd1	115.1 (3)	S22^i^—Pd2—S25^i^	87.77 (4)
C19—N10A—Pd1	117.4 (3)	S25—Pd2—S25^i^	180.0
C9—N10A—H10A	103.2	C23—S22—Pd2	109.52 (14)
C19—N10A—H10A	103.2	N24—C23—S22	175.1 (4)
Pd1—N10A—H10A	103.2	C26—S25—Pd2	107.53 (16)
C9—N10B—C19	117.7 (11)	N27—C26—S25	176.9 (5)
C9—N10B—Pd1	115.0 (8)		
			
C5—N1—N2—C3	0.8 (4)	C15—N11—N12—C13	0.6 (4)
Pd1—N1—N2—C3	173.4 (2)	Pd1—N11—N12—C13	179.3 (3)
C5—N1—N2—C8	−178.9 (3)	C15—N11—N12—C18	−172.6 (3)
Pd1—N1—N2—C8	−6.4 (4)	Pd1—N11—N12—C18	6.1 (4)
N1—N2—C3—C4	−0.9 (4)	N11—N12—C13—C14	−0.1 (5)
C8—N2—C3—C4	178.8 (4)	C18—N12—C13—C14	172.2 (4)
N1—N2—C3—C6	178.5 (4)	N11—N12—C13—C16	−178.4 (4)
C8—N2—C3—C6	−1.7 (7)	C18—N12—C13—C16	−6.1 (7)
N2—C3—C4—C5	0.6 (4)	N12—C13—C14—C15	−0.4 (5)
C6—C3—C4—C5	−178.8 (5)	C16—C13—C14—C15	177.7 (5)
N2—N1—C5—C4	−0.4 (4)	N12—N11—C15—C14	−0.8 (5)
Pd1—N1—C5—C4	−171.2 (3)	Pd1—N11—C15—C14	−179.3 (3)
N2—N1—C5—C7	179.1 (3)	N12—N11—C15—C17	177.5 (4)
Pd1—N1—C5—C7	8.3 (5)	Pd1—N11—C15—C17	−1.0 (7)
C3—C4—C5—N1	−0.1 (4)	C13—C14—C15—N11	0.8 (5)
C3—C4—C5—C7	−179.6 (4)	C13—C14—C15—C17	−177.4 (5)
C3—N2—C8—C9	−115.1 (4)	C13—N12—C18—C19	−113.4 (4)
N1—N2—C8—C9	64.6 (4)	N11—N12—C18—C19	58.3 (4)
N2—C8—C9—N10B	−75.9 (12)	C9—N10A—C19—C18	166.9 (4)
N2—C8—C9—N10A	−42.8 (5)	Pd1—N10A—C19—C18	30.0 (6)
N10B—C9—N10A—C19	−63.7 (12)	C9—N10A—C19—N10B	58.3 (11)
C8—C9—N10A—C19	−165.4 (4)	Pd1—N10A—C19—N10B	−78.6 (10)
N10B—C9—N10A—Pd1	74.3 (11)	N12—C18—C19—N10A	−77.7 (5)
C8—C9—N10A—Pd1	−27.4 (6)	N12—C18—C19—N10B	−46.7 (11)
N10A—C9—N10B—C19	68.0 (18)	C9—N10B—C19—N10A	−72.5 (18)
C8—C9—N10B—C19	160.4 (10)	Pd1—N10B—C19—N10A	64.0 (11)
N10A—C9—N10B—Pd1	−67.2 (12)	C9—N10B—C19—C18	−156.5 (11)
C8—C9—N10B—Pd1	25.2 (17)	Pd1—N10B—C19—C18	−20.0 (15)

Symmetry code: (i) −*x*+1, −*y*+2, −*z*+2.

### Hydrogen-bond geometry (Å, º)

**Table:**

*D*—H···*A*	*D*—H	H···*A*	*D*···*A*	*D*—H···*A*
N10*A*—H10*A*···N24^ii^	0.90	2.01	2.889 (9)	166
N10*B*—H10*B*···S1^iii^	0.90	2.71	3.52 (2)	151

Symmetry codes: (ii) *x*, *y*, *z*−1; (iii) *x*−1, *y*, *z*.
